# Fluctuations in Medium Viscosity May Affect the Stability of the CAG Tract in the *ATXN2* Gene

**DOI:** 10.3390/biomedicines12102396

**Published:** 2024-10-19

**Authors:** Anna Dorohova, Oksana Lyasota, Stepan Dzhimak, Alexandr Svidlov, Olga Leontyeva, Mikhail Drobotenko

**Affiliations:** 1 Laboratory of Problems of Stable Isotope Spreading in Living Systems, Federal Research Center the Southern Scientific Center of the Russian Academy of Sciences, 344006 Rostov-on-Don, Russia; 4098789@mail.ru (O.L.); jimack@mail.ru (S.D.); svidlov@mail.ru (A.S.); 2 Research Department, Kuban State University, 350040 Krasnodar, Russia; 89002926536ola@gmail.com (O.L.); mdrobotenko@mail.ru (M.D.)

**Keywords:** *ATXN2* gene, CAG tract, trinucleotide repeat expansion diseases, DNA mathematical model, torque

## Abstract

**Background:** Trinucleotide repeats are the cause of many neurodegenerative diseases that are currently incurable. In this regard, the question of the causes of occurrence and methods of prevention or treatment of diseases caused by the expansion of repeats in the CAG tract of the *ATXN2* gene remains relevant. Previously, it was shown that the frequency of occurrence of additional OS (open states) zones increases with increasing length of the CAG tract, and the value inverse to the frequency correlates with the age of disease onset. **Methods:** In this work, the influence of the viscosity of the medium and the external torque on the stability of the CAG tract in the *ATXN2* gene was studied using mathematical modeling methods. **Results:** It has been established that the probability of the appearance of additional OS zones of significant size increases with an increase in the CAG of the tract (k > 40 CAG repeats) for all viscosity values, however, at k ≤ 40, the change in viscosity does not significantly affect the probability of additional OS zones in the tract. **Conclusions:** It was found that under normal conditions (absence of pathology), viscosity does not have a reliable effect on the stability of the DNA molecule, but when pathology appears, an increase in viscosity contributes to an increase in DNA stability, and, accordingly, a decrease has a negative effect on the stabilization of the DNA molecule. In the zone of close to incomplete penetrance of the disease, viscosity does not have a reliable effect on the stability of the CAG tract.

## 1. Introduction

Numerous studies have associated the *ATXN2* gene [1] with a wide range of neurodegenerative diseases. In healthy individuals, the *ATXN2* polyQ tract typically consists of 21–22 CAG repeats [2,3]. Expansion of the CAG tract by 34 or more trinucleotide repeats is a genetic cause of spinocerebellar ataxia type II (SCA 2) and other neurodegenerative diseases [4,5,6,7,8]. Intermediate polyQ expansions in the 27–33 CAG range were found to be a significant risk factor for 4.7% of ALS cases [9]. SCA2 is a progressive motor neuron disease characterized by the death of cerebellar neurons [10]. In contrast to predominantly cerebellar disorders, the SCA2 variant can develop quite rapidly into multisystem neurodegeneration with early involvement of the brainstem, spinal cord, midbrain (sometimes manifesting as parkinsonism) and muscles (seizures) [11,12]. The mechanisms responsible for increasing the length of the CAG tract have not yet been identified [13,14]; some authors associate them with non-canonical configurations in the CAG tract that arise during repair, transcription and replication processes [15,16,17,18]. The above processes are characterized by unwinding of duplexes, and therefore the DNA is subjected to torsional stress [19,20], causing the chains to diverge. In separated DNA chains, regions containing CAG repeats can form secondary structures (for example, hairpins), which affect the reading of genetic information [21,22,23].

In [24], a connection has been established between the stability of the CAG tract and its length (the number of CAG repeats). The stability of the CAG tract is understood as the probability of the additional open states zones genesis in the CAG tract under torque. It has been shown that the genesis frequency of additional OS zones increases with the length of the CAG tract, and the value inverse to the frequency correlates with the age of onset of the disease. Of particular interest is the study of the influence of external factors on the stability of the CAG tract, one of which is the viscosity of the surrounding medium. Viscosity has previously been shown to play an important stabilizing role in DNA dynamics [25].

It is known that pronounced changes in the viscosity of the nucleoplasm are one of the most important mechanisms for the development of certain diseases, for example, Alzheimer’s and Parkinson’s diseases, due to changes in the dynamics of transport of biomolecules and organelles inside the nucleus. These processes can also be accompanied by disruptions in the functioning of the genetic apparatus [26,27,28].

The purpose of this work is to study the influence of environmental viscosity on the stability of the CAG tract in the *ATXN2* gene using mathematical modeling methods.

## 2. Mathematical Model

To study the stability of the CAG tract in the *ATXN2* gene, we will use the angular model of the DNA molecule [29,30], which is based on the analogy between a double-stranded DNA molecule and a mechanical system consisting of two chains of interconnected pendulums, and is a system of ordinary differential equations with respect to the angular deviations of the pendulums [31]:

(1) $$I_{1}^{i}\frac{d^{2}\varphi_{1}^{i}\left(t\right)}{dt^{2}}=K_{1}^{i}\left[\varphi_{1}^{i-1}\left(t\right)-2\varphi_{1}^{i}\left(t\right)+\varphi_{1}^{i+1}\left(t\right)\right]-\;\delta^{i}\left(k_{12}^{i}R_{1}^{i}\left(R_{1}^{i}+R_{2}^{i}\right)sin\varphi_{1}^{i}+k_{12}^{i}R_{1}^{i}R_{2}^{i}\sin\left(\varphi_{1}^{i}-\varphi_{2}^{i}\right)\right)+F_{1}^{i}\left(t\right),\;\;i=\bar{2,n-1},$$

(2) $$I_{1}^{1}\frac{d^{2}\varphi_{1}^{1}\left(t\right)}{dt^{2}}=K_{1}^{1}\left[\varphi_{1}^{2}\left(t\right)-\varphi_{1}^{1}\left(t\right)\right]-\;\delta^{i}\left(k_{12}^{1}R_{1}^{1}\left(R_{1}^{1}+R_{2}^{1}\right)\sin\varphi_{1}^{1}+k_{12}^{1}R_{1}^{1}R_{2}^{1}\sin\left(\varphi_{1}^{1}-\varphi_{2}^{1}\right)\right)+F_{1}^{1}\left(t\right),$$

(3) $$I_{1}^{n}\frac{d^{2}\varphi_{1}^{n}\left(t\right)}{dt^{2}}=K_{1}^{n}\left[\varphi_{1}^{n-1}\left(t\right)-\varphi_{1}^{n}\left(t\right)\right]-\;\delta^{i}\left(k_{12}^{n}R_{1}^{n}\left(R_{1}^{n}+R_{2}^{n}\right)\sin\varphi_{1}^{n}+k_{12}^{n}R_{1}^{n}R_{2}^{n}\sin\left(\varphi_{1}^{n}-\varphi_{2}^{n}\right)\right)+F_{1}^{n}\left(t\right),$$

(4) $$I_{2}^{i}\frac{d^{2}\varphi_{2}^{i}\left(t\right)}{dt^{2}}=K_{2}^{i}\left[\varphi_{2}^{i-1}\left(t\right)-2\varphi_{2}^{i}\left(t\right)+\varphi_{2}^{i+1}\left(t\right)\right]+\;\delta^{i}\left(k_{12}^{i}R_{2}^{i}\left(R_{1}^{i}+R_{2}^{i}\right)\sin\varphi_{2}^{i}-k_{12}^{i}R_{1}^{i}R_{2}^{i}\sin\left(\varphi_{2}^{i}-\varphi_{1}^{i}\right)\right)+F_{2}^{i}\left(t\right),\;\;i=\bar{2,n-1},$$

(5) $$I_{2}^{1}\frac{d^{2}\varphi_{2}^{1}\left(t\right)}{dt^{2}}=K_{2}^{1}\left[\varphi_{2}^{2}\left(t\right)-\varphi_{2}^{1}\left(t\right)\right]+\;\delta^{i}\left(k_{12}^{1}R_{2}^{1}\left(R_{1}^{1}+R_{2}^{1}\right)\sin\varphi_{2}^{1}1-k_{12}^{1}R_{1}^{1}R_{2}^{1}\sin\left(\varphi_{2}^{1}-\varphi_{1}^{1}\right)\right)+F_{2}^{1}\left(t\right),$$

(6) $$I_{2}^{n}\frac{d^{2}\varphi_{2}^{n}\left(t\right)}{dt^{2}}=K_{2}^{n}\left[\varphi_{2}^{n-1}\left(t\right)-\varphi_{2}^{n}\left(t\right)\right]+\;\delta^{i}\left(k_{12}^{n}R_{2}^{n}\left(R_{1}^{n}+R_{2}^{n}\right)\sin\varphi_{2}^{n}-k_{12}^{n}R_{1}^{n}R_{2}^{n}\sin\left(\varphi_{2}^{n}-\varphi_{1}^{n}\right)\right)+F_{2}^{n}\left(t\right).$$

Here,

$\varphi_{j}^{i}\left(t\right)$—angular deviation of the *i*-pendulum of the *j*-chain, counted counterclockwise, at time *t*;

$I_{j}^{i}$—moment of inertia of the *i*-pendulum of the *j*-chain;

$R_{j}^{i}$—distance from the center of mass of the *i*-pendulum of the *j*-chain to the thread;

$K_{j}^{i}$—constant characterizing the torque of the *i*-section of the *j*-thread;

$k_{12}^{i}$—constant characterizing the elastic properties of the connection of the *i*-pair of pendulums;

$F_{j}^{i}\left(t\right)$—external influence on the *i*-pendulum of the *j*-chain at time *t*.

n—the number of pairs of pendulums in the system.

The values of the Equations (1)–(6) coefficients were obtained experimentally and described in the work [29]. In Equations (1)–(6), the first term to the right of the equal sign describes the force action on the *i*-th pendulum from the elastic thread, the second term from the paired pendulum, the third term the external force action. The magnitude of the external action $F_{j}^{i}\left(t\right)=-\beta_{j}^{i}\frac{d\varphi_{j}^{i}}{dt}\left(t\right)+M\left(t\right)$, where the term $-\beta_{j}^{i}\frac{d\varphi_{j}^{i}}{dt}\left(t\right)$ models the dissipation effects caused by the interaction with the liquid medium around the DNA molecule [32], the term $M^{i}\left(t\right)$ is the torque [32]. By changing the parameter λ, we will simulate the change in the viscosity of the medium surrounding the DNA molecule.

Equations (1)–(6) allow us to describe the hydrogen bond in the *i*-th pair ($\delta^{i}=1$) and the breaking of this bond ($\delta^{i}=0$) [33]. We assume that a break occurs in the *i*-th base pair if the potential energy of the bond in this pair exceeds the critical value *E_AT_* for the AT pair and *E_GC_* for the GC pair; the bond is restored if its potential energy becomes less than the critical value [34]. The energy values for hydrogen bonds breaking in AT and GC pairs are taken from [25].

To Equations (1)–(6), we add the initial conditions:

(7) $$\varphi_{1}^{i}\left(0\right)=0,\frac{d\varphi_{1}^{i}}{dt}\left(0\right)=0,$$

(8) $$\varphi_{2}^{i}\left(0\right)=\pi ,\frac{d\varphi_{2}^{i}}{dt}\left(0\right)=0,i=\bar{1,n},$$

which define the unperturbed initial state.

Torque $M^{i}\left(t\right)$ was chosen to be constant in time and spatially localized on the segment [*i*_1_, *i*_2_], i.e.,

$$M^{i}\left(t\right)=M_{0}^{i},\;i=\bar{1,n}$$

Moreover, $M_{0}^{i}$ = $M_{0}$ at 1 ≤ *i*_1_ ≤ *i* ≤ *i*_2_ ≤ n and $M_{0}^{i}$ = 0 for other i values.

During the study, the system of Equations (1)–(8) was solved numerically by the fourth-order Runge–Kutta method using an original program written by the authors of the article [35].

## 3. Results

As in [24], to minimize computational costs when solving the problem (1)–(8), not the entire *ATXN2* gene was selected, but only the region containing 23 CAG repeats from the 4601st to the 6600th base pairs. This region contains the first exon of the *ATXN2* gene. The region was selected taking into account that when exposed to the torque over the calculated time interval, the disturbance zone does not reach the boundaries of the selected region, which allows for adequate boundary conditions. Note that with the expansion of the CAG tract, the right border of the selected area increased accordingly.

For the viscosity parameter *λ* = 1.0, calculations showed that at M_0_ = 8.28 pN · nm, OS zones appear in the promoter region. With an increase in the M_0_ value, additional OS zones of various sizes can form in the CAG tract [24]. The probability of OS zones occurrence characterizes the stability of the CAG tract.

**Figure 1:**
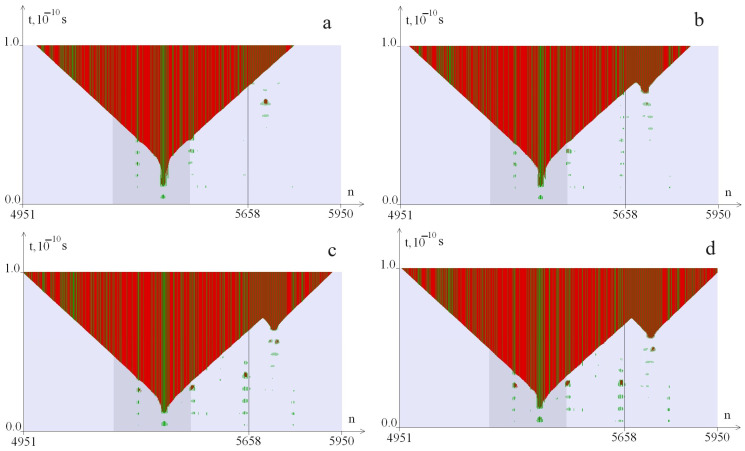
Dynamics of the OS zones genesis from the torque influence with localization on the segment (650, 1320) of the *ATXN2* gene region at k = 40, i_2_ = 1195, *λ* = 0.9. OS in AT pairs are shown in green, in GC pairs—in red. The promoter region is highlighted with a darker background. (**a**) corresponds to M_0_ = 8.33 pN·nm; (**b**)—M_0_ = 8.31 pN·nm; (**c**)—M_0_ = 8.27 pN·nm; (**d**)—M_0_ = 8.56 pN·nm. The numbers of nitrogenous base pairs are marked horizontally (n), and time is marked vertically (t).

**Figure 2:**
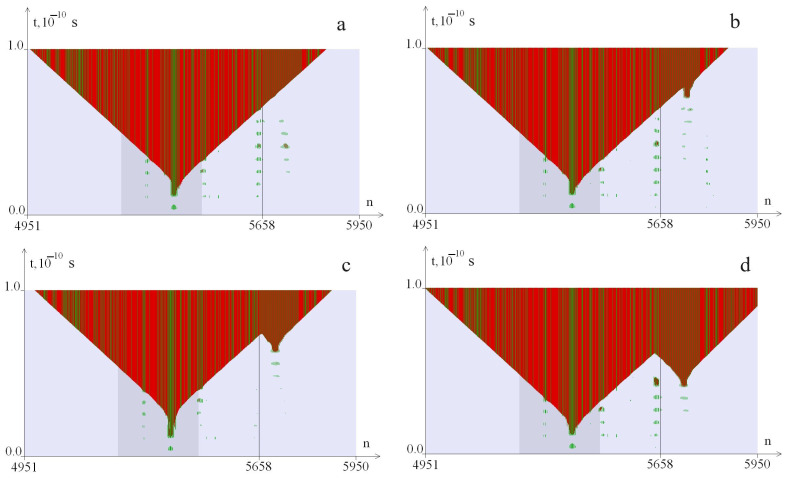
Dynamics of the OS zones genesis from the torque influence with localization on the segment (650, 1320) of the *ATXN2* gene region at k = 40, i_2_ = 1205, *λ* = 1.0. OS in AT pairs are shown in green, in GC pairs—in red. The promoter region is highlighted with a darker background. (**a**) corresponds to M_0_ = 8.40 pN·nm; (**b**)—M_0_ = 8.45 pN·nm; (**c**)—M_0_ = 8.29 pN·nm; (**d**)—M_0_ = 8.48 pN·nm. The numbers of nitrogenous base pairs are marked horizontally (n), and time is marked vertically (t).

**Figure 3:**
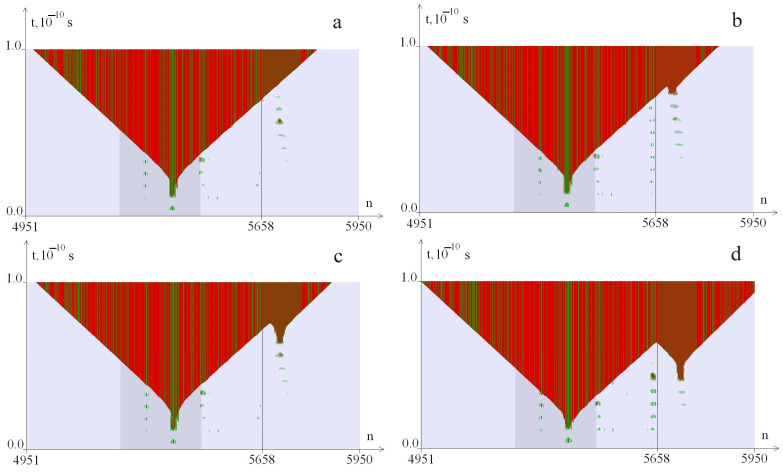
Dynamics of the OS zones genesis from the torque influence with localization on the segment (650, 1320) of the *ATXN2* gene region at k = 40, i_2_ = 1210, *λ* = 1.1. OS in AT pairs are shown in green, in GC pairs—in red. The promoter region is highlighted with a darker background. (**a**) corresponds to M_0_ = 8.33 pN·nm; (**b**)—M_0_ = 8.35 pN·nm; (**c**)—M_0_ = 8.32 pN·nm; (**d**)—M_0_ = 8.49 pN·nm. The numbers of nitrogenous base pairs are marked horizontally (n), and time is marked vertically (t).

As a result of the numerical experiment, we obtained 32,550 images, which were analyzed according to the following criteria, described using the example of Figure 1, Figure 2 and Figure 3. Figure 1 shows examples of the additional OS zones genesis at a CAG tract length of k = 40 (*λ* = 0.9) at different values of the torque. Figure 2 shows examples of the additional OS zones genesis at a CAG tract length of k = 40 (*λ* = 1.0) at different values of the torque. Figure 3 shows examples of the additional OS zones genesis at a CAG tract length of k = 40 (*λ* = 1.1) at different values of the torque. The green color in the figure indicates the OS in AT pairs; the red color indicates the OS in GC pairs. The promoter region is highlighted with a darker background; the line (5641st base pair) indicates the beginning of the CAG tract. The numbers of nitrogenous base pairs are marked horizontally (n), and time is marked vertically (t). The Figure 1 shows that at M_0_ = 8.27 pN·nm, an OS zone appears in the promoter zone (Figure 1a, Figure 2a and Figure 3a); with an increase in the M_0_ value, additional OS zones may appear in the CAG tract. Additional zones of insignificant size (Figure 1b, Figure 2b and Figure 3b) are not taken into account in the analysis of the CAG tract stability; large zones (Figure 1c,d, Figure 2c,d and Figure 3c,d) are considered significant and are taken into account in the analysis of the CAG tract stability.

**Figure 4:**
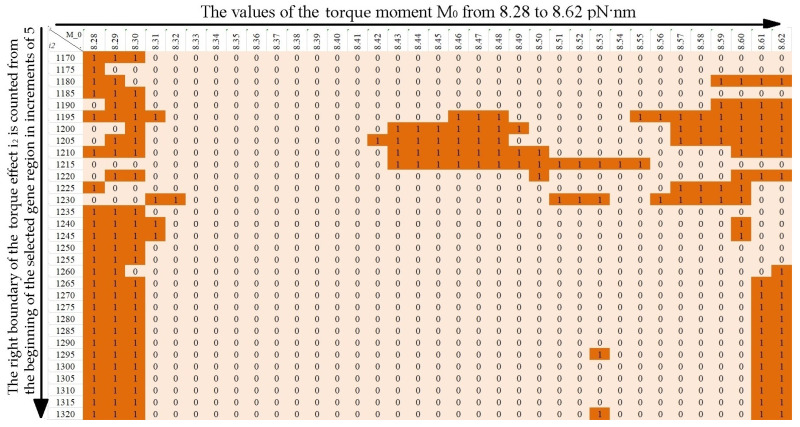
Additional OS zones for k = 40, *λ* = 0.9.

**Figure 5:**
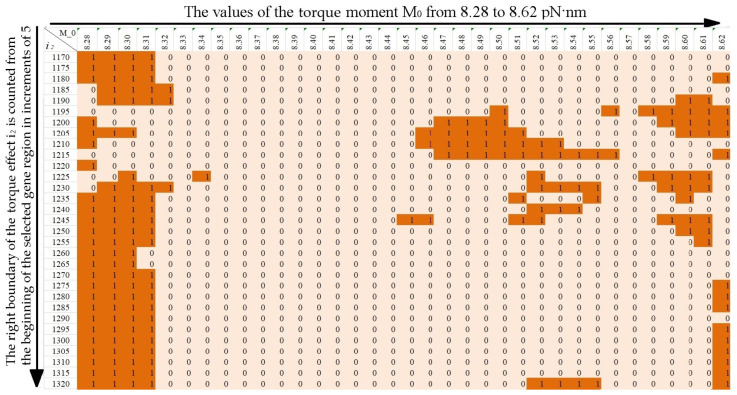
Additional OS zones for k = 40, *λ* = 1.0.

**Figure 6:**
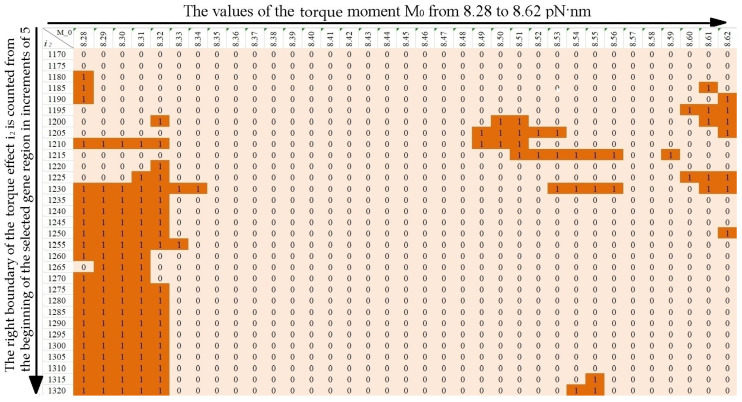
Additional OS zones for k = 40, *λ* = 1.1.

To analyze the effect of viscosity on the stability of the CAG tract, calculations were carried out for values of the viscosity parameter *λ* = 0.9, *λ* = 1.0 and 1.1 at the length of the CAG tract k = 30, 32, 34, 35, 40, 45, 50, 55, 60, 65. Torque values were chosen to be the same as in the [24]. The Figure 4, Figure 5 and Figure 6 show horizontally the values of the torsion moment M_0_, vertically the right boundary of the torsion action i_2_ counted from the beginning of the selected section of the gene. The left boundary of the torque localization is equal to the left boundary of the promoter region, i.e., the 650th base pair from the beginning of the selected region of the gene.

The values in the Figure 4, Figure 5 and Figure 6 characterize the size of additional OS zones, where 0 means that the additional OS zone is absent or has an insignificant size, and 1 means that the additional OS zone has a significant size. For clarity, the cells of the figures containing 1 are additionally highlighted in color (an examples are in Figure 4, Figure 5 and Figure 6 for k = 40, *λ* = 0.9, *λ* = 1.0 and *λ* = 1.1).

Using the data from the figures, the probabilities of additional OS zones occurrence of significant size in the CAG tract were calculated.

Note that a change in the viscosity parameter of the medium leads to a change in the minimum value of the torque required for the OS zone genesis in the promoter region (M_0_ = 8.27 pN·nm for *λ* = 0.9, M_0_ = 8.29 pN·nm for *λ* = 1.1), but for consistency, for all values of the parameter *λ*, the probability calculation was carried out in the range M_0_ from 8.28 to 8.62 pN·nm.

**Figure 7:**
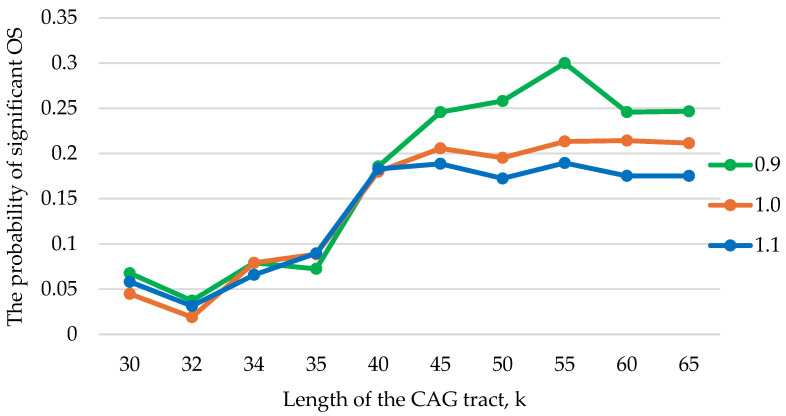
Graphs of the probability of significant size additional OS zones genesis in the CAG tract for different values of the viscosity parameter *λ*.

Graphs of the probability dependence of additional OS zones genesis on the length of the CAG tract for different values of the viscosity parameter *λ* are shown in Figure 7.

## 4. Discussion

Most biochemical and biophysical reactions in living systems occur in an aquatic environment. The speed and nature of these reactions depend on a number of factors [36,37]. The enthalpy and entropy of DNA and proteins also largely depend on the properties of the aquatic environment [38,39,40]. In addition, the localization of energy in the DNA molecule and the free energy of interactions between nitrogenous bases also depend on environmental parameters [41,42,43,44].

Since viscosity plays a stabilizing role in DNA dynamics, it consequently affects the formation of OS zones. Numerical experiments have shown that at k > 40 for the viscosity value *λ* = 0.9, an increase in the number of additional OS zones is observed relative to *λ* = 1.0, and at *λ* = 1.1, a decrease occurs.

It should be noted that in addition to the viscosity of the medium, other factors can influence the stability of the CAG tract, such as localization of CAA interruptions [45].

Figure 7 shows that if the CAG tract length does not exceed 40 (k ≤ 40), then the change in viscosity does not significantly affect the number of additional OS zones that arise in the tract. This may be due to the fact that 32–36 CAG repeats correspond to the zone of incomplete penetrance of the disease [46]. This threshold effect suggests that the number of CAG tract repeats 35–40 is likely to be the turning point that converts normal polyQ proteins into pathogenic ones [47,48].

It is known that one of the mechanisms for the implementation of the trinucleotide repeat diseases effects are secondary structures of DNA (for example, hairpins, G-quadruplex, R-loop, triplex DNA) [49,50,51,52], which can form when additional OS zones arise in the CAG tract. The obtained data indicate about the formation of additional large OS zones in the region of the CAG tract, which makes it possible to form secondary structures in it. Moreover, a decrease in the viscosity of the medium surrounding the DNA molecule leads to an increase in the probability of additional OS zones genesis with a pathological number of CAG repeats, and an increase in viscosity leads to a decrease in their number. Some groups of drugs used in drug therapy of the neurodegenerative diseases and symptomatic treatment of their clinical manifestations can change blood viscosity [53,54,55]. It is unknown whether the viscosity of the medium inside the cell nuclei containing DNA changes, but such studies of the viscosity influence on the OS genesis undoubtedly have potential for practical application.

It is known that the dissipation of mechanical energy of the DNA molecule in a viscous medium ensures its stability [32,56,57]. Viscous interaction of water molecules with the negatively charged phosphate backbone of DNA is possible because they are hydrogen bond donors [58,59]; in addition, water molecules can form hydrogen bonds with each other [43,60,61,62,63].

At this stage of development, the angular DNA model does not allow separating the phases of the aqueous environment. When carrying out calculations in this work, the generalized viscosity of the medium surrounding the DNA molecule was taken into account. The strong point of the model is the ability to carry out calculations of the effect of viscosity on the dynamics of open states in individual gene regions, which correlates with the results of the work [64]. The viscous medium is at the same time the most important factor limiting the possible types of conformational movements, which is fundamentally important from the point of view of limiting the choice of possible trajectories during the folding of the macromolecule [65,66,67]. In [68], it was noted that at all stages of spatial structure formation, a clear correlation of rotations by torsion angles is observed. With an increase in the viscosity of the medium, the duration of the process increases proportionally. Our results are completely consistent with these conclusions.

## 5. Conclusions

A decrease in the viscosity of the medium below critical values leads to a change in the dynamics of the macromolecule, and significant conformational changes occur [68].

Numerical experiments have proven that the occurrence of OS in a DNA molecule depends, among other things, on the viscosity of the external environment, and not only on the magnitude of the torque and the length of the CAG tract. It has been established that the probability of additional OS zones occurrence of significant size increases at k > 40 for all viscosity values, from which it can be concluded that under normal conditions (absence of pathology), viscosity does not have a reliable effect on the stability of the DNA molecule, but when pathology appears, an increase in viscosity contributes to an increase in DNA stability, and, accordingly, a decrease has a negative effect on the stabilization of the DNA molecule. In addition, an increase in viscosity leads to a change in the minimum of the torque value required to open the promoter region.

## Data Availability

Data are contained within the article.
